# Personal

**Published:** 1911-11

**Authors:** 


### PERSONAL

Dr. James H. Glass of Utica, has purchased 200 acres in Herkimer Co., on the east side of West Canada Creek, at Trenton Falls.

Dr. Smith Baker of Utica has an article in the *Popular Science Monthly* on the Significance of Abdominal Pain.

Dr. J. G. Kilbourn of Utica entertained the staff of St. Luke's Hospital and a few other local physicians at his summer home in the outskirts of New Hartford, on September 23, 1911. Trap and rifle shooting, a base ball game and a clam bake occupied the afternoon and evening.

Dr. Charles E. Flagg of Buffalo spent part of September at the Lake of Bays, Ontario.

Dr. Jesse E. Levy of Buffalo had a night call,, October 1, from a couple of burglars. They showed presence of mind, on being detected, by claiming to be sick but, as they were trying to enter by the window instead of the door, Dr. Levy declined to consider the call of a professional nature.

Dr. Charles A. Brownell and Dr. F. S. Hoffman of Buffalo, returned about the middle of October from the Bermudas.

Dr. Edward E. Haley, of Buffalo, Chairman of the Committee on Contract Practice of the Medical Society of the County of Erie, himself entered into a life contract on October 3, 1911. Our congratulation and best wishes.

Dr. Herbert B. Smith of Corning and Dr. Ira Goff of Howard, are the Republican candidates for coroners of Steuben County.

Dr. William Gaertner of Buffalo, is the Democratic candidate for Superintendent of Education of the City of Buffalo, in opposition to Henry P. Emerson, LL.D., who has occupied the position for nearly twenty years. The editor was a pupil of the latter, a teacher of the former. Both are splendid men, both have been professional teachers, in high schools. We are glad that a medical journal does not have to take sides in a political issue.

---

Dr. Maud J. Frye has returned from Europe.

---

Dr. B. D. Shedd of Arcade, spent part of October in the Adirondacks, hunting deer.

---

Dr. Alexander Hugh Ferguson of Chicago, announces a partnership with his former first assistant, Dr. James J. Monahan, with offices in the Reliance Building, 32 N. State St. Chicago.

---

Dr. L. Duncan Bulkley will give the thirteenth series of Clinical Lectures on Diseases of the Skin at the New York Skin and Cancer Hospital, Second Avenue and 19th Street, New York City, Wednesdays, November 1 to December 20, 1911, at 4.15 P. M. The course is free to members of the medical profession.

---

Dr. George A. Himmelsbach of Buffalo has returned from Europe.

---

The Medical Society of the County of Steuben held its 94th fall meeting at Corning, October 10, 1911, electing Dr. John L. Miller of Corning, President; Dr. J. A. Conway of Hornell, Vice-President and re-electing Dr. W. W. Smith of Avoca, Secretary and Treasurer.

---

Dr. Howard A. Kelly, of Baltimore, Md., and Ahmic Lake, Ont., had to break in upon his recent vacation and have a troublesome chole cystitis treated surgically. He has made an excellent recovery.

---

Dr. Herman E. Hayd of Buffalo, has returned from the meeting of the American Association of Obstetricians and Gynecologists, of which he has been President.

---

Dr. Julius T. Cohen of Buffalo, has returned from Europe.

---

Dr. W. A. Scott of Niagara Falls, has been renominated by the Republicans for a fourth term as Coroner of Niagara County.

Dr. W. G. Grove of Buffalo, spent several months last summer touring the British Islands and western Europe.

Dr. Bernard F. Dennis, after four years of European study, has opened an office at 520 Franklin St., Buffalo, for the practice of surgery, having especially qualified for cystoscopy, etc.

